# Wing-feather loss in white-feathered laying hens decreases pectoralis thickness but does not increase risk of keel bone fracture

**DOI:** 10.1098/rsos.220155

**Published:** 2022-06-15

**Authors:** Renée Garant, Bret W. Tobalske, Neila Ben Sassi, Nienke van Staaveren, Tina Widowski, Donald R. Powers, Alexandra Harlander-Matauschek

**Affiliations:** ^1^ Department of Animal Biosciences, University of Guelph, 50 Stone Road E, Guelph, ON N1G 2W1, Canada; ^2^ Division of Biological Sciences, University of Montana, 32 Campus Drive, Missoula, MT 59812, USA; ^3^ Department of Biology, George Fox University, 414N Meridian Street, Newberg, OR 97132, USA

**Keywords:** feather loss, flapping flight, muscle adaptations, bird, keel bone damage

## Abstract

Feather loss in domestic chickens can occur due to wear and tear, disease or bird-to-bird pecking. Flight feather loss may decrease wing use, cause pectoral muscle loss and adversely impact the keel bone to which these muscles anchor. Feather loss and muscle weakness are hypothesized risk factors for keel bone fractures that are reported in up to 98% of chickens. We used ultrasound to measure changes in pectoral muscle thickness and X-rays to assess keel bone fracture prevalence following symmetric clipping of primary and secondary feathers in white- and brown-feathered birds. Four and six weeks after flight feather clipping, pectoralis thickness decreased by approximately 5%, while lower leg thickness increased by approximately 5% in white-feathered birds. This pectoralis thickness decrease may reflect wing disuse followed by muscle atrophy, while the increased leg thickness may reflect increased bipedal locomotion. The lack of effect on muscle thickness in brown-feathered hens was probably due to their decreased tendency for aerial locomotion. Finally, pectoralis thickness was not associated with keel bone fractures in either white- or brown-feathered birds. This suggests that the white-feathered strain was more sensitive to feather loss. Future prevention strategies should focus on birds most susceptible to muscle loss associated with flight feather damage.

## 1. Introduction

Flying birds need relatively large flight muscles. To physically accommodate these muscles, birds have a protrusion of the sternum called the keel bone. Directly attaching to this bone is the pectoralis, a muscle that inserts onto the humerus and pulls the wing down during a downstroke. The antagonist, the supracoracoideus, attaches to the keel and lifts the wing by its tendon, creating a pulley over the coracoid to connect with the humerus and generate an upstroke [1]. Together, these muscles produce the forces necessary for flight. These energy-consuming flight muscles are capable of rapid changes in size and composition in response to environmental cues, such as feed intake and temperature, or during ageing, moulting or migratory events [2–4]. To illustrate, sequential ultrasound measurements of the pectoral muscles showed that changes to muscle thickness could occur rapidly, parallel with body mass decrease, and even within a 10 h endurance flight in migratory birds [5].

Many species within the Phasianidae family, including domestic chickens (*Gallus gallus domesticus*) kept for egg-laying, are heavy birds with strong leg muscles [6]. These birds are found predominantly on the ground walking, running, jumping and scratching. They have limited flight capabilities due to naturally high wing loading [7]. León *et al*. [8] showed that laying hens descend at a velocity of nearly 4 m s^−1^, two to three times greater than adept fliers [9–11], meaning they have poor flight control. However, flight control may be further challenged by reducing wing area through natural feather moult, feather loss and damage due to bird-to-bird pecking or abrasions [12]. Bird-to-bird pecking occurs in 15–95% of backyard chickens and birds kept on farms and can target any feathered area [12–14]. This can include the wing feathers, though the extent with which wing feathers are affected is flock dependent [12,15,16]. However, severe bird-to-bird pecking can result in featherless hens over time [13,17]. Regardless of its cause, a reduction to the wing area leads to increased wing loading and could reduce flight capabilities [8], which may put laying hens at an increased risk of collisions and so contribute to keel fractures.

Collisions are often assumed as a probable source of keel fractures. This has been supported by studies which demonstrate increased rates of keel bone fracture in hens housed in aviaries compared with hens in caged systems [18–22]. However, rates of keel bone fracture of cage-housed hens are still reported at rates of approximately 15–55% despite these hens lacking the space to collide with structures [18,22,23].

More recently, Thøfner *et al*. [24] showed that high-energy collisions were unlikely to be a main cause of keel fractures. Instead, it was suggested that a majority of fractures probably originate from within the bird, which has been speculated to result from increased egg-laying, loading patterns on the bone, or forces applied on the bone from the flight muscles [24,25]. A currently unexplored area of research that may explain the origins of keel fracture is the activity of the flight muscles (pectoralis and supracoracoideus). Hens housed in cages have limited space to move and exercise their flight muscles, which has been shown to contribute to low leg and wing bone density [26,27] and reduced weight of the flight muscles [28]. On the contrary, it has been shown that laying hens given the opportunity to exercise have a greater bone density of their keel, tibiae, humeri and radii [29–32]. Laying hens with increased wing loading spent less time using their wings to access aerial resources [33]. Strains of laying hens with increased wing loading were found to have fewer keel bone fractures and deviations than strains with lower wing loading [34]. As the flight muscles are large and place tremendous force onto the keel bone [35], it seems likely that inactive hens would have a weaker keel bone. The weaker keel bone can then be subject to fracture during bouts of vigorous wing flapping, a common occurrence during fear/panic responses [7] or slips from perches [36] and platforms.

Longitudinal investigations into the muscle and bone development in relation to keel bone fractures have been limited due to the need to sacrifice birds to conduct measurements. With recent advances in technology, there are now less invasive ways to objectively measure keel bone fractures through radiography [37–39] and muscle thickness using ultrasonography [40,41] over time. Muscle thickness is shown to decrease or increase in humans undergoing periods of muscle disuse or training [42,43], respectively. In chickens, muscle thickness is correlated with breast muscle weight and size [44]. To the best of the authors’ knowledge, no one has investigated the relationship between flight restriction and flight muscle use and thickness in relation to keel bone fractures in chickens.

Therefore, in the present study, we used a symmetric reduction in wing area to mimic damaged wing feathers and restrict flight in brown- and white-feathered chickens. We predicted that progressive increases in wing loading due to feather clipping would cause an incremental atrophy of the flight muscle, while hypertrophy was expected in the lower leg muscles due to reduced aerial and increased ground locomotion. Finally, we assumed birds with greater wing loading were more likely to show keel fractures due to forces on the keel from a higher wing flapping frequency, a behavioural mechanism to compensate for wing area loss, and the resultant internal strain would produce fractures of the keel.

## 2. Material and methods

### 2.1. Animals and housing

A total of 120 female domestic chickens of two strains (white-feathered aged 34 weeks, average weight of 1765 ± 26 g; brown-feathered aged 39 weeks, average weight of 2090 ± 26 g) were used in the experiment. Brown-feathered birds were reared under commercial management conditions in enriched aviary housing until 23 weeks of age and white-feathered birds in enriched colony housing until 18 weeks of age. Upon transfer to the laying facility birds were divided into 12 identical floor pens (183 L × 244 W × 290 H cm). Each pen housed 10 hens (one strain per pen) and each hen was fit with an individually numbered identification ‘backpack’ made with two elastic straps attached via eyelets to a soft silicone rectangle (14.5 × 6 × 0.2 cm) [45]. Pens were bedded with wood shavings and located in a ventilated, windowless room (14 : 10 L : D cycle) at the Research Station of the University of Guelph (Guelph, ON). Each pen was fixed with two identical slatted platforms (122 L × 31 W × 70 H cm) and a perch attached to the long side of one platform (122 L at 70 H cm). A second perch was the highest point in the pen and spanned the width of the pen (244 L at 150 H cm). Hens had access to feed in two 5 kg hanging feeders (Frandsen Corporation, North Branch, Minnesota, USA) and two nest-boxes: one on the ground and the second atop a platform, and water from 12 nipple drinkers. White opaque boards were placed along adjoining pen walls to prevent contact and social learning from hens of neighbouring pens. Hens were habituated to the experimental set-up for approximately 17 weeks before start of the experiment. Hens were maintained on a commercial management plan under 4–6 lux. Temperature of the barn was recorded prior to and during the trial. In the four weeks leading up to the trial's start date, the barn temperature averaged approximately 24°C (max. 27.3°C), and during the trial, the average temperature was approximately 21°C (max. 23.2°C).

### 2.2. Flight feather clipping treatment

Beginning on week 0, after baseline measures of muscle thickness (see below) had been acquired, hens received one of three wing clipping treatments to both wings as per Garant *et al*. [33]. Treatments were applied to randomly selected birds within each pen in a way that each of the three treatments was represented within each pen (approx. three birds/treatment/pen). In brief, treatments included clipping of the primary and secondary flight feathers (full-clip; 55.4% reduction to wing area), clipping of the primary flight feathers (half-clip; 32.5% reduction to wing area) and no clipping (unclipped; 0% reduction to wing area) [8,33]. The flight feather clipping technique was adapted from Harrison & Flinchum [46] and involved clipping the flight feathers along the edge of the coverts with sterile scissors, paying special attention to avoid nicking newly forming blood feathers [47].

### 2.3. Muscle thickness

**Figure 1.**
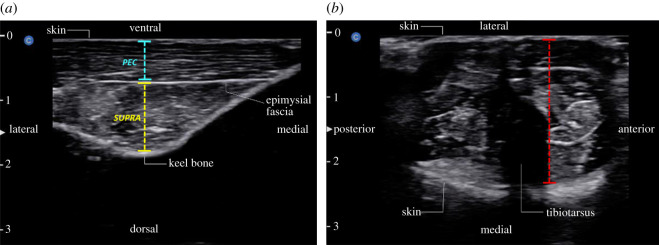
Ultrasound images of (*a*) the pectoralis (PEC), supracoracoideus (SUPRA) and keel bone and, (*b*) lower leg (the cumulative measurement of *M. gastrocnemius pars medialis, M. fibularis lateralis* and *M. tibialis cranialis caput tibiale *and* femorale*). Each example ultrasound image is labelled with anatomical landmarks and anatomical planes as well as ticked lines to show where thickness measurements were acquired for each muscle measured. Ticked numbers to the left of each image indicate the imaging depth of the linear transducer in cm.

A portable ultrasound machine (EBit 50 Unit, Digital Colour Doppler Ultrasound System, Chison Medical Technologies, Bellevue, WA, USA) was used to obtain cross-sectional images of the breast (*pectoralis* and *supracoracoideus*) and lower leg muscles (*M. gastrocnemius pars medialis*, *M. fibularis lateralis* and *M. tibialis cranialis caput tibiale *and* femorale*) on week 0 (baseline, before clipping treatment), and two, four and six weeks after clipping application. Images were obtained for the left and right breast and lower leg muscles. To obtain images, each hen was gently restrained by hand and placed on their backs on the lap of an assistant. Feathers were gently parted (not detached) to expose the skin, and multipurpose ultrasound gel (Wavelength Ultrasound Gel (blue), National Therapy Products, Brampton, ON, Canada) was used as a contact agent. A high-frequency 7.0–18.0 MHz linear transducer was used to capture images. Transducer placement was adapted from Dietz *et al*. [48] to ensure consistent placement to obtain comparable results. The flat end of a plastic vernier calliper was placed at the cranial apex of the keel, lying perpendicular to its length to form a 90° angle. The transducer was placed caudal to the calliper and perpendicular to the keel, and the image was captured. The pectoralis was measured from below the skin to the epimysial fascia, and the supracoracoideus was measured from just below the epimysial fascia to the keel bone (figure 1).

To ensure consistent transducer placement when imaging the lower legs, each hen was gently restrained and placed laterally on their left (for left leg) or right side (for right leg) in the lap of an assistant. The assistant would gently hold both feet such that the shank and lower leg formed a 90° angle. With the leg in this position, the flat end of a plastic vernier calliper was placed just below the knee and perpendicular to the tibiotarsus. The transducer was placed caudal to the calliper. Lower leg thickness measurements included the cumulative measurement of the *M. gastrocnemius pars medialis*, *M. fibularis lateralis* and *M. tibialis cranialis caput tibiale *and* femorale*. Thickness was measured between the skin of the lateral and medial sides of the lower leg (figure 1).

Images were transferred from the ultrasound system to a computer where ImageJ software was used to obtain muscle thickness measurements (cm) [49]. The person who analysed the images was unaware of the treatments. Left and right muscle measurements were then averaged to give one measure of thickness per muscle measured (pectoralis, supracoracoideus and lower leg) for each time point of data collection (week 0, 2, 4 and 6).

### 2.4. Bodyweight and keel bone fractures

The weight of each hen (kg) was recorded on week 0 (baseline), and two, four and six weeks after treatment application. X-ray imaging was used to determine if keel bone fractures were present. Digital radiographs were obtained with a Poskom VET-20BT portable X-ray unit (Promark Imaging, Toronto, ON, Canada) on week 0 (baseline measure) and six weeks after clipping, as adapted from Woolcott *et al*. [50]. Imaging was conducted inside a steel-doored concrete room at the Research Station of the University of Guelph. A custom-made wooden box fixed with evisceration shackles was used to position hens for imaging laterally. The wooden box contained a built-in add-on for a wireless DD imagine receptor panel (dimensions: 29 × 24 × 31 mm, weight: 3.4 kg). The X-ray unit was suspended approximately 80 cm from the receptor panel as each image was taken (image matrix of 19.65 × 23.6 cm, resolution of 77 μm @2560 × 3072 pixels). A built-in crank allowed the height of the panel to be adjusted depending on hen size to allow proper alignment of the panel with the keel bone. The legs of each hen were gently placed into the shackles, and hens remained in an upside-down position for approximately 10 s as the image was taken [39]. The image quality and image density parameters of the X-ray unit were 90 kVp and 20 mAs, respectively. As the receptor panel had a 2.4 and 5.0 GHz wireless gigabit interface, radiographs were directly stored onto a wirelessly connected computer as images were taken.

**Figure 2.**
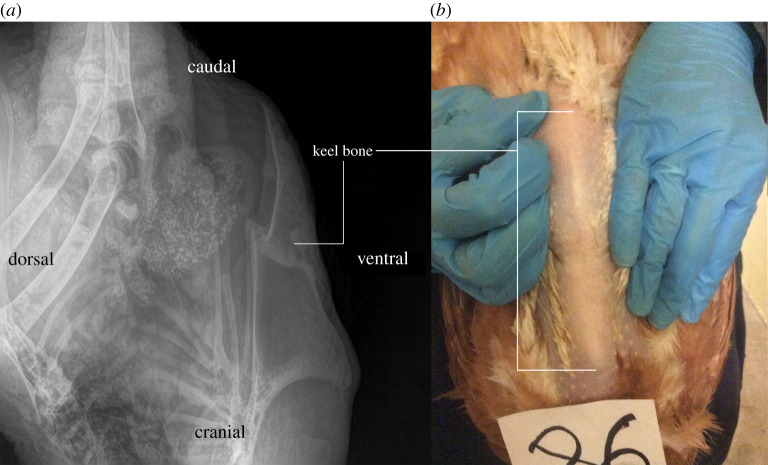
Images of an adult laying hen with a fractured keel bone. (*a*) a radiograph of a lateral view of the keel bone labelled with anatomical planes and, (*b*) a photograph of a frontal view of the keel bone and the surrounding skin, photo includes ID number.

After obtaining radiographs (figure 2), images were scored by a trained observer unaware of the treatments for the presence and severity of keel bone fractures based on online tutorials and instruction provided by Rufener *et al*. [51]. In brief, keel fracture severity was assessed on a 0–5 scale according to severity. A score of ‘0’ indicated no fracture while a score of ‘5’ indicated extremely severe fracture (fracture(s) affecting a large area of bone; often with dislocated segments). A score greater than 0 was used to assign the presence or absence (score 0) of keel bone fracture in the birds.

### 2.5. Statistical analysis

Due to missing images and injurious pecking that occurred throughout the trial, nine brown-feathered birds were removed from the keel bone data (3 full-clip, 2 half-clip and 4 unclipped) leaving a final 111 birds. Similarly, for the muscle thickness and bodyweight data analysis this meant that two brown-feathered hens were removed from the analysis on week 0 (2 unclipped), five hens by week 2 (2 full-clip, 1 half-clip and 2 unclipped); seven hens by week 4 (2 full-clip, 1 half-clip, 4 unclipped); and 11 hens by week 6 (4 full-clip, 3 half-clip and 4 control). As such, the final number of birds included in muscle thickness/bodyweight analysis was 118 (0 weeks), 115 (2 weeks), 113 (4 weeks) and 109 (6 weeks).

Change in muscle thickness was calculated as the difference between muscle thickness at time of measurements (two, four and six weeks after clipping) and baseline (week 0) expressed as a percentage of baseline muscle thickness with the following equation:

$$\left(\frac{\left(\mathrm{muscle}\;\mathrm{thicknes}s_{\mathrm{week}\;x}-\mathrm{muscle}\;\mathrm{thicknes}s_{\mathrm{week}\;0}\right)}{{\mathrm{muscle}\;\mathrm{thickness}}_{\mathrm{week}\;0}}\right)\times 100.$$

The percentage change in bodyweight was calculated in a similar manner.

Analyses of percentage change in muscle thickness were conducted with a generalized linear mixed model procedure (PROC GLIMMIX) in SAS v. 9.4 (SAS Institute Inc., Cary, NC, USA, 2012) for the pectoralis, supracoracoideus and lower leg muscles. Each model evaluated the percentage change in muscle thickness in comparison with the baseline (week 0) using fixed effects of flight feather clipping treatment (control, half-clip and full-clip), strain (white-feathered and brown-feathered) and their interaction. A similar calculation and analysis was conducted for the percentage change in bodyweight. Least squared means (LSM) and standard error (s.e.) are presented to compare the percentage change between clipping treatments over time compared with baseline (week 0) within each strain unless stated otherwise. Additionally, we evaluated whether the percentage change was significantly different from zero.

The relationship of keel bone fracture presence and percentage change of the pectoralis muscle six weeks after clipping was analysed with a generalized linear model procedure (PROC GLIMMIX) in SAS v. 9.4. The model evaluated the percentage change in pectoralis muscle thickness six weeks after clipping using fixed effects of fracture presence score (0: no fracture, greater than 0: fracture present) and bird strain (white-feathered and brown-feathered) and their interaction.

Normally distributed residuals and homogeneity of variance were assessed with studentized residual plots, and the distribution of best fit was determined with the Shapiro–Wilk statistic. A Kenward–Roger approximation was used to calculate degrees of freedom, and a Tukey–Kramer adjustment was used to account for multiple comparisons. Results were considered statistically significant at a *p*-value of <0.05.

Keel bone fracture presence was categorized as 0 (no fracture) and 1 (fracture present, score >0). Analysis of fracture presence was conducted with a generalized linear model procedure (PROC GLIMMIX) in SAS v. 9.4 for binary outcomes. The odds of having fractures (0,1) were modelled using week (week 0, week 6), flight feather clipping treatment (control, half-clip and full-clip), and strain (white-feathered and brown-feathered), and their interactions as the fixed effects. Results are presented as odds ratio and 95% confidence interval where an odds ratio greater than 1 indicates a higher risk of keel bone fracture. Due to a small proportion of birds having severe scores, the keel bone fracture severity was not further analysed (score 0: 51.4%, score 1: 27.9%, score 2: 14.4%, score 3: 5.4%, score 4: 0.9%, score 5: 0.0%).

## 3. Results

Muscle thickness (mm) values of the pectoralis, supracoracoideus, lower leg muscles, as well as bodyweight (g) from week 0 are presented in table 1 and the percentage change of these variables in table 2. The percentage change is shown over time (two, four and six weeks after clipping compared with week 0) within white- and brown-feathered birds for each clipping treatment. A positive percentage change indicates that the muscle was thicker than at week 0, while a negative percentage change indicates a thinner muscle then at week 0.

**Table 1.** Descriptive statistics (mean, standard deviation (s.d.), minimum (min) and maximum (max)) for muscle thickness (mm), as measured by ultrasound, and bodyweight (g) in white- and brown-feathered birds at baseline (week 0), *N* is the number of birds. Measured muscles include the pectoralis, supracoracoideus and lower leg (cumulative measurement of the *M. gastrocnemius pars medialis*, *M. fibularis lateralis*, and *M. tibialis cranialis caput tibiale *and* femorale*).

muscle	treatment	white-feathered				
		*N*	mean	s.d.	min	max
pectoralis	unclipped	24	5.15	0.42	4.40	6.32
	half-clipped	18	5.32	0.53	4.56	6.75
	full-clipped	18	5.33	0.42	4.62	6.11
supracoracoideus	unclipped	24	10.74	0.48	9.59	11.71
	half-clipped	18	10.96	0.48	10.30	11.93
	full-clipped	18	10.93	0.57	9.92	11.92
lower leg	unclipped	24	21.44	1.36	19.02	23.68
	half-clipped	18	21.56	1.31	18.72	23.42
	full-clipped	18	21.73	1.37	19.45	23.89
bodyweight	unclipped	24	1777	148	1524	2130
	half-clipped	18	1828	145	1630	2140
	full-clipped	18	1825	116	1634	2060
		brown-feathered				
muscle	treatment	*N*	mean	s.d.	min	max
pectoralis	unclipped	22	4.85	0.45	4.10	5.64
	half-clipped	18	4.94	0.49	4.18	5.74
	full-clipped	18	4.93	0.49	3.85	5.82
supracoracoideus	unclipped	22	11.29	0.89	9.90	13.60
	half-clipped	18	11.51	1.07	9.89	13.42
	full-clipped	18	11.32	0.60	10.05	12.48
lower leg	unclipped	22	26.41	1.72	22.65	29.75
	half-clipped	17	26.65	2.01	22.01	29.41
	full-clipped	18	26.46	1.99	22.65	29.19
bodyweight	unclipped	22	2076	173	1846	2546
	half-clipped	18	2124	144	1924	2400
	full-clipped	18	2089	150	1830	2466

**Table 2.** Changes (%) LSM ± standard errors of the mean (s.e.) in pectoralis, supracoracoideus and lower leg muscle thickness (cumulative measurement of the *M. gastrocnemius pars medialis*, *M. fibularis lateralis*, and *M. tibialis cranialis caput tibiale *and* femorale*) as measured by ultrasound, and bodyweight in white- and brown-feathered hens two, four and six weeks after wing feather damage (unclipped, half-clipped and full-clipped). Within a given muscle group and certain week, LSM ± s.e. that do not share a letter superscript indicates that the percentage change from baseline/week 0 is significantly different between clipping treatments (*p* < 0.05). Values that are italics show a percentage of change that is significantly different from baseline.

muscle	treatment	weeks since treatment		
		2 weeks	4 weeks	6 weeks
*white-feathered*				
pectoralis	unclipped	*13.20 ± 3.09%*^A^	2.97 ± 3.44%^A^	1.70 ± 2.06%^A^
	half-clipped	*7.77 ± 3.34%*^A^	−5.10 ± 3.61%^B^	*−5.78 ± 2.32%*^B^
	full-clipped	*7.24 ± 3.34%*^A^	−4.86 ± 3.61%^B^	*−6.01 ± 2.32%*^B^
supracoracoideus	unclipped	−1.36 ± 1.61%^A^	−2.34 ± 1.33%^A^	2.51 ± 1.13%^A^
	half-clipped	−1.91 ± 1.71%^A^	−2.78 ± 1.43%^A,B^	2.14 ± 1.27%^A^
	full-clipped	−3.37 ± 1.71%^A^	*−5.84 ± 1.43%*^B^	0.49 ± 1.27%^A^
lower leg	unclipped	*7.79 ± 1.65%*^A^	*5.88 ± 1.86%*^A^	*9.04 ± 1.95%*^A^
	half-clipped	*5.30 ± 1.78%*^A^	*5.63 ± 1.97%*^A^	*6.44 ± 2.08%*^A^
	full-clipped	*3.73 ± 1.78%*^A^	3.52 ± 1.97%^A^	*7.00 ± 2.08%*^A^
bodyweight	unclipped	0.40 ± 1.12%^A^	0.26 ± 1.27%^A^	0.28 ± 1.36%^A^
	half-clipped	*−3.10 ± 1.16%*^B^	*−4.79 ± 1.33%*^B^	*−4.45 ± 1.43%*^B^
	full-clipped	*−4.86 ± 1.16%*^B^	*−6.28 ± 1.33%*^B^	*−6.37 ± 1.43%*^B^
*brown-feathered*				
pectoralis	unclipped	*18.63 ± 3.16%*^A^	*13.15 ± 3.55%*^A^	*5.99 ± 2.22%*^A^
	half-clipped	*9.85 ± 3.40%*^B^	5.13 ± 3.66%^B^	1.85 ± 2.51%^A^
	full-clipped	*10.99 ± 3.48%*^A,B^	6.59 ± 3.71%^A,B^	3.25 ± 2.60%^A^
supracoracoideus	unclipped	*−3.58 ± 1.64%*^A^	*−3.51 ± 1.39%*^A^	2.52 ± 1.22%^A^
	half-clipped	*−3.91 ± 1.73%*^A^	*−5.00 ± 1.45%*^A^	−0.77 ± 1.38%^A^
	full-clipped	*−4.94 ± 1.76%*^A^	*−4.02 ± 1.48%*^A^	0.60 ± 1.43%^A^
lower leg	unclipped	2.02 ± 1.69%^A^	2.14 ± 1.93%^A^	−1.03 ± 2.03%^A^
	half-clipped	1.84 ± 1.85%^A^	1.90 ± 2.03%^A^	2.53 ± 2.24%^A^
	full-clipped	2.57 ± 1.86%^A^	1.60 ± 2.04%^A^	3.67 ± 2.24%^A^
bodyweight	unclipped	1.00 ± 1.13%^A^	1.90 ± 1.30%^A^	2.93 ± 1.40%^A^
	half-clipped	−0.77 ± 1.17%^A^	−1.90 ± 1.34%^A^	−1.52 ± 1.49%^A^
	full-clipped	−0.32 ± 1.19%^A^	−0.94 ± 1.36%^A^	0.21 ± 1.52%^A^

### 3.1. Pectoralis

Two weeks after clipping, all hens, regardless of strain or treatment group, showed a significant increase (7% to 19%) in pectoralis thickness (table 2, all *p* < 0.05). Within the white-feathered birds, this increase in muscle thickness did not differ between the differently clipped groups. However, considering the brown-feathered strain, the unclipped hens showed the largest increase in muscle thickness, nearly double the increase in the half-clip group (*t*_99.6_ = 2.51, *p* = 0.0366). The full-clipped birds showed an intermediary increase that did not differ from the half-clipped (*t*_99.3_ = 0.30, *p* = 0.9507) or the unclipped birds (*t*_100.5_ = 2.13, *p* = 0.0891; table 2).

Four weeks after clipping, the thickness increases observed two weeks post-treatment had reduced such that there was no significant change from week 0 for nearly all clipping groups of both strains. Only the brown-feathered unclipped hens maintained a significant thickness increase of over 10% (*t*_97.55_ = 2.59, *p* = 0.0292) which differed from the lesser increase of the half-clipped birds (6.59 ± 3.71%, *t*_97.11_ = 2.70, *p* = 0.0223).

While the white-feathered strain showed no difference between clipping groups at two weeks, at four weeks after clipping the unclipped birds started to differ from the half-clipped (*t*_97.1_ = 2.78, *p* = 0.0178) and full-clipped (*t*_97.1_ = 2.70, *p* = 0.0223). The pectoralis muscle became numerically thinner compared with week 0 in half- and full-clipped birds, while it stayed numerically thicker compared with week 0 in unclipped birds. Consequently, both the half- and full-clipped birds showed a relative decrease in thickness compared with the unclipped birds where muscle remained thicker.

Six weeks after clipping, the pectoralis thickness increase observed two weeks post-clipping declined further such that now the half- (*t*_42.6_ = −2.50, *p* = 0.0165) and full-clipped (*t*_42.6_ = −2.60, *p* = 0.0129) white-feathered birds had significantly thinner muscles compared with week 0. By contrast, the unclipped, white-feathered birds had the same muscle thickness at six weeks as at week 0 (table 2). Therefore, half-clipped and full-clipped birds had significantly thinner pectoralis muscles compared with the unclipped group (*t*_92.2_ = 2.65, *p* = 0.0254; *t*_92.2_ = 2.73, *p* = 0.0076, respectively).

In the brown-feathered birds, no differences were observed any more between clipping treatments at week 6. However, unclipped, brown-feathered hens were the only ones to maintain a significant thickness increase compared with week 0 (*t*_36.7_ = 2.70, *p* = 0.0105).

### 3.2. Supracoracoideus

Two weeks after clipping, the supracoracoideus was less thick compared with week 0 regardless of clipping treatment; however, this decrease was only significant in the brown-feathered birds (all *p* > 0.05, table 2). No differences were observed between unclipped, half-clipped and fully clipped birds within either the white- or brown-feathered strain (table 2). The supracoracoideus decrease for the brown-feathered strain remained four weeks after clipping in all treatment groups (all *p* > 0.05).

For the white-feathered strain, the supracoracoideus was nearly 6% thinner after four weeks than before clipping in the full-clip group (*t*_22.88_ = −4.10, *p* = 0.0004), while no change was observed in the unclipped and half-clipped birds. This decrease in thickness was significantly different from the numerical decrease in the white-feathered unclipped group (*t*_97.3_ = 2.62, *p* = 0.0270), but similar to the numerical decrease seen in the half-clipped group (table 2). Six weeks after clipping, the supracoracoideus was of similar thickness as in week 0 in both strains, and no differences were found between birds with different flight feather clipping.

### 3.3. Bodyweight and lower leg muscles

There were no significant changes to bodyweight or lower leg muscle thickness two, four and six weeks after clipping was applied for the brown-feathered strain. Furthermore, the clipping status of the brown-feathered birds did not lead to differences in percentage change of bodyweight or lower leg muscle thickness at any of the time points (table 2).

In white-feathered birds, the lower leg muscles became generally thicker compared with week 0 (all *p* < 0.05), with the only exception in full-clipped birds at four weeks (table 2). While the muscles became thicker (approx. 4% to 9%), this increase in muscle thickness was the same in unclipped, half-clipped and full-clipped birds (table 2). Interestingly, the half- and full-clipped birds also showed a long-lasting decrease in bodyweight (3% to 6.5% decrease) that remained noticeable throughout the trial (2-, 4- and 6-week post-clipping). Their bodyweight was consistently lower than what it had been at week 0, while the unclipped birds remained at the same bodyweight as at week 0. This showed a significantly higher percentage change in the unclipped birds’ bodyweight than the half- and full-clipped birds (table 2).

### 3.4. Keel bone fractures

The percentage of birds with keel bone fractures at baseline (week 0) and six weeks after clipping are presented in table 3. Fracture rates were identical between white- and brown-feathered birds at baseline and six weeks after flight feather clipping. Nearly all white-feathered (92.9%) and brown-feathered hens (80.8%) had a mild fracture (score 1 or 2) six weeks after treatment application; only 6.31% of hens had a moderate-severe fracture (score 3 or 4), and none of the hen's keels had a severe fracture (score of 5). Overall, fracture rate of the entire flock (*n* = 111) was 48.6% by the trial's end.

**Table 3.** OR estimates and 95% CI of having a keel bone fracture. An OR > 1 indicates that birds were more likely to have a keel bone fracture, whereas an OR < 1 indicates that birds were less likely to have a keel bone fracture. The number (*n*) and percentage (%) of laying hens with a keel bone fracture at baseline (week 0) and six weeks after wing damage (unclipped, half-clipped and full-clipped) in white- and brown-feathered birds of two strains (white- and brown-feathered), and of the three treatment groups are included.

treatment	*n* (%)	OR	95% CI
*week*			
week 0	46 (41.4%)	ref.	ref.
week 6	54 (48.6%)	1.43	0.82–2.48
*strain*			
brown	47 (46.1%)	ref.	ref.
white	53 (44.2%)	0.9	0.57–1.73
*clipping*			
unclipped	39 (44.3%)	ref.	ref.
half-clipped	36 (52.9%)	1.4	0.73–2.65
full-clipped	25 (37.9%)	0.71	0.36–1.41

On week 0, 46 hens had at least one keel bone fracture (41.4% flock prevalence). This included 21 brown-feathered and 25 white-feathered hens. Six weeks after clipping, 54 hens (26 brown-feathered and 28 white-feathered) were found to have a keel bone fracture giving a 48.6% flock prevalence. The odds of having a keel bone fracture at six weeks after clipping was not different compared with before clipping was applied (week 0: 41.4%, week 6: 48.6%, OR = 1.43, 95% CI 0.82–2.48). Similarly, the odds of having keel bone fractures did not differ between white-feathered (44.2%) and brown-feathered hens (46.1%, OR = 0.9, 95% CI 0.57–1.73). Half-clipped (52.9%) and fully clipped (37.9%) birds were not more likely to have keel bone fractures compared with unclipped birds (44.3%; OR = 1.4, 95% CI 0.73–2.65 (half), OR = 0.71, 95% CI 0.36–1.41 (full), respectively). Furthermore, there were no significant differences between treatment groups within strains (table 4).

**Table 4.** OR estimates and 95% CI of the number (*N*) and percentage (%) of birds with three levels of wing damage (unclipped, half-clipped and full-clipped) within a strain (white- and brown-feathered) with a keel bone fracture. An OR > 1 indicates that birds were more likely to have a keel bone fracture, whereas an OR less than 1 indicates that birds were less likely to have a keel bone fracture.

strain	clipping status	week	*N* (%)	OR	95% CI
white-feathered	unclipped	week 0	9 (37.5%)	ref.	ref.
		week 6	10 (41.7%)	1.2	0.36–3.91
	half-clipped	week 0	9 (50.0%)	ref.	ref.
		week 6	10 (55.6%)	1.3	0.32–4.87
	full-clipped	week 0	7 (38.9%)	ref.	ref.
		week 6	8 (44.4%)	1.3	0.32–4.98
brown-feathered	unclipped	week 0	10 (50.0%)	ref.	ref.
		week 6	10 (50.0%)	1	0.28–3.60
	half-clipped	week 0	8 (50.0%)	ref.	ref.
		week 6	9 (56.3%)	1.3	0.30–5.48
	full-clipped	week 0	3 (20.0%)	ref.	ref.
		week 6	7 (46.7%)	3.5	0.64–19.06

### 3.5. Relationship between keel bone fracture presence and pectoralis thickness six weeks post-treatment

Within the brown-feathered strain, birds with a keel bone fracture (score > 0) had a pectoralis thickness increase of 4.51 ± 2.13% six weeks after clipping. This thickness increase did not differ from brown-feathered birds without a fracture (score 0) (3.36 ± 2.16%; *t*_98.5_ = −0.42, *p* = 0.6767). Similarly, white-feathered birds with a fracture had a muscle thickness decrease of −1.52 ± 2.05% that did not differ from birds without fracture (−4.03 ± 1.94%, *t*_103.5_ = −1.00, *p* = 0.3197).

## 4. Discussion

Wing feathers are susceptible to damage through wear-and-tear, disease and bird-to-bird pecking. As such, we mimicked wing feather damage through symmetric flight feather clipping and subsequently used ultrasonography to study its effects on adaptive changes in pectoral and leg muscles in two strains of adult laying hens over a period of six weeks. We further hypothesized that internal muscle forces on the already fragile keel bone would increase the presence of keel fractures as a result of adaptive behavioural changes (increase in wing flapping frequency) used to compensate for wing feather damage.

Regardless of damaged or undamaged wing feathers, pectoralis thickness was increased compared with baseline in white- and brown-feathered birds two weeks after treatment. This was surprising. In wild birds, flight muscle thickness increases during cold acclimatization [52–54] as they are a primary thermogenic organ [55–57]. Theoretically, a temperature drop in the barn after baseline could lead to pectoralis muscle fibre contractions, shivering, generating heat as part of a negative feedback mechanism of maintaining body temperature [58–60]. There was a seasonal temperature drop that coincided with the week we began our baseline measurements. In the four weeks leading up to the trial's start date, the barn temperature averaged at approximately 24°C (max. 27.3°C) but during the trial the average temperature was approximately 21°C (max. 23.2°C). Numerous studies have reported that the growth of chickens is optimum at lower temperatures of 18–21°C and declines at temperature values nearing or greater than 30°C [61–64]. While temperature may have affected observed changes in pectoralis muscle thickness, the barn remained near 21°C for the duration of the trial. Theoretically, observed thickness increases should have been maintained at this temperature rather than increasing two weeks post-clipping, and declining to values similar to baseline by week 6.

Damaged wing feathers (half- and full-clipped birds) were associated with pectoralis thickness and bodyweight decreases four and six weeks after clipping in white-feathered hens. Most of the flapping-flight power comes from the downstroke generated by the pectoralis as wings move down and forward [65]. The loss in pectoral muscle thickness may reflect reduced muscle protein synthesis due to reduced flapping-flight activity induced by feather damage (particularly in the fully clipped birds) [33], similar to skeletal muscle loss caused by inactivity in other species of birds [66,67]. In addition, there is a connection between bodyweight and muscle mass in birds [5,68], which can explain the similar relationship found for pectoralis thickness and bodyweight changes. Garant *et al*. [33] showed that damaged feathers in white-feathered laying hens reduced elevated feeder usage. Potentially, this could lead to reduced feed intake and muscles may have been catabolized to divert protein and energy to egg production; however, birds still had ad libitum access to feed through the ground feeder. Moreover, the feather loss, which represents a 3.98% loss in bodyweight, may have been a psychological stressor by limiting the birds’ freedom of movement to access a previously used resource (elevated feeder) and possibly increasing competition (ground feeder). This could potentially trigger a loss in appetite, reduced feed intake and increased protein degradation of the pectoralis muscles, thereby, exacerbating muscle loss. Unfortunately, we were unable to measure individual feed intake in the current study, but this should be considered in future experiments.

Interestingly, brown-feathered birds with undamaged wings showed an increased pectoralis thickness at the end of the trial. This could potentially be explained by the fact that birds with undamaged wings spent more than two times as much time at elevated resources than brown-feathered birds with damaged (half-clipped and fully clipped) wings [33]. This could indicate that changes in pectoralis muscle thickness require power-training in brown-feathered birds, similar to the effects of muscle training in other species [69,70]. It is unclear why the effect of clipping treatment showed as a reduction in muscle thickness in white-feathered birds (disuse; half/fully clipped) but as an increase in muscle thickness in brown-feathered birds (use; unclipped). Potentially, this difference could be explained through the fact that brown-feathered birds naturally are more ground-dwelling while white-feathered birds are more aerial [71–74].

In general, brown-feathered birds were less affected by feather damage as bodyweight, supracoracoideus thickness and lower leg muscle thickness did not change over time after treatment. The maintenance of the thickness of these muscles and bodyweight suggests that the ground-dwelling behaviour of brown-feathered birds makes it easier to cope with wing feather damage. It is also possible that the supracoracoideus, which produces minimal aerodynamic power [68], is not as heavily relied upon as the pectoralis for aerial locomotion in brown-feathered birds. Brown-feathered chickens may use their powerful legs to jump up more so than relying on the strong upstroke of the supracoracoideus (personal observations). By contrast, when they descend, they may flap more vigorously, activating the pectoralis for the downstroke, to reduce the descent velocity of their heavy bodies. This could potentially explain why stronger changes occurred to the pectoralis compared with the supracoracoideus.

All white-feathered hens saw a significant increase in lower leg muscle thickness two and six weeks after the start of the trial. These increases could hypothetically be attributed to increased ground locomotion. However, as the birds with undamaged and damaged wings showed a similar increase this is probably not the case. Instead, it could be that the leg muscle thickness in all groups was simply increasing over time. The fact that the half- and fully clipped birds, however, showed small decreases in their bodyweight compared with week 0 suggests that the reduction in bodyweight in these birds did indeed not come from the loss of leg muscles but from the previously described loss of pectoralis muscles.

Birds in this study were somewhat heavier than the published target weight according to the breed-specific guidelines. White-feathered birds were 110 g heavier than the target weight at the start of the trial (34 weeks of age) [75]. Thus, while white-feathered birds with damaged wing feathers did lose approximately 5% of their bodyweight by the trial's end, they were still within their target weight. Similarly, the brown-feathered birds also weighed approximately 184 g more than their target weight [76]. Small changes in bodyweight over the time period of the trial were to be expected [75,76]. The fact that most of our birds (except for the half- and fully clipped white-feathered birds) did not show a change in bodyweight over the course of the trial could be due to different factors including the fact that our birds were already heavier than projected.

Fracture rates in our study were identical between white- and brown-feathered birds at baseline and six weeks after flight feather clipping (table 3). The overall keel fracture rate was similar to the 35–65% reported by other studies of hens aged 30–50 weeks of age from various housing systems [18,77–79]. Most of these fractures were considered mild. Perhaps, if clipping had been applied at a younger age when hens were most active, there may have been a more prominent influence of treatment on keel fracture presence and severity. It is noteworthy to point out that 81.7% of fractures were present in the caudal area of the keel bone (data not shown), similar to results of Casey-Trott *et al*. [78] and Thøfner *et al*. [24]. This region is the last to fully ossify at 35–40 weeks of age [80], and fractures in this area are thought to be mostly from internal factors rather than collisions. Internal factors of bird strain, age or feather damage and the consequent changes in muscle thickness did not influence keel fractures in the current study, suggesting that other internal factors could play a role. It would have been interesting to relate our results to individual egg production as this is related to keel bone fractures [81]. However, Darwin noted a high level of keel deformation back in 1868 when documenting the physical characteristics of chickens [82] that had not yet undergone selection for egg production. In summary, there is more to keel bone fracture development than we currently understand, and this highlights the need for continued research.

Finally, there are some limitations to this study that should be acknowledged. First, we used wing feather clipping as a tool to mimic feather damage and restrict flight in a home pen environment. However, it should be noted that this does not restrict birds in their ability to use their wings (stretching, flapping, etc.) and a quick trip up to a perch or platform may not be rigorous enough to incite any muscle changes. This could explain the limited differences observed between the clipping treatments. More restrictive immobilization techniques could have limited wing use further and allowed for larger differences in muscle thickness or keel bone fractures to be observed. Additionally, while experimental pens were organized to mimic characteristics present in commercial aviaries (littered floor area, platforms and elevated resources), we acknowledge that the experimental pens do not fully reflect a commercial aviary, (greater number and density of birds), which may have affected the response to feather clipping. Second, we used ultrasonography which allowed us to non-invasively track changes in muscle thickness of the same hens over several weeks. However, the technology is limited as muscles are three-dimensional structures, and changes in mass can occur along multiple dimensions. For keel bone fracture, a measure of keel bone density could be added in future studies to determine the degree to which flight muscle activity affects keel strength in adult hens in addition to the fracture outcome itself. However, a measure of keel bone density requires sacrifice of the birds, which limits longitudinal measurement on the same bird which was the aim of this study. Lastly, we tested one strain for each hen colour; therefore, we cannot overgeneralize our findings to all white- or brown-feathered hens. Future studies should include multiple strains of each colour to tease out strain effects.

## 5. Conclusion

We found evidence that feather damage induced by clipping of flight feathers alters the morphology of a strain of white-feathered laying hens, through a significant decrease to bodyweight and pectoralis thickness. By contrast, the strain of brown-feathered hens was not affected by feather damage, possibly due to their more ground-oriented mobility. Additionally, the strain, feather damage and subsequent muscle thickness, or week post-treatment were not associated with the presence of keel bone fracture. This implies that other untested factors probably function in the development of keel bone fracture. Further investigations into the causes of keel bone fractures are warranted because of the implications for animal welfare and rearing economics.

## Data Availability

Raw data have been made publicly available as electronic supplementary material [83].
